# An author keyword analysis for mapping Sport Sciences

**DOI:** 10.1371/journal.pone.0201435

**Published:** 2018-08-01

**Authors:** Luis-Millán González, Xavier García-Massó, Alberto Pardo-Ibañez, Fernanda Peset, José Devís-Devís

**Affiliations:** 1 Departamento de Educación Física y Deportiva, Universidad de Valencia, Valencia, Spain; 2 Departamento de Didáctica de la Expresión Musical, Plástica y Corporal, Universidad de Valencia, Valencia, Spain; 3 Instituto Universitario de Matemática Pura y Aplicada, Universitat Politècnica de València, Valencia, Spain; Institut Català de Paleoecologia Humana i Evolució Social (IPHES), SPAIN

## Abstract

Scientific production has increased exponentially in recent years. It is necessary to find methodological strategies for understanding holistic or macro views of the major research trends developed in specific fields. Data mining is a useful technique to address this task. In particular, our study presents a global analysis of the information generated during last decades in the Sport Sciences Category (SSC) included in the Web of Science database. An analysis of the frequency of appearance and the dynamics of the Author Keywords (AKs) has been made for the last thirty years. Likewise, the network of co-occurrences established between words and the survival time of new words that have appeared since 2001 has also been analysed. One of the main findings of our research is the identification of six large thematic clusters in the SSC. There are also two major terms that coexist ('REHABILITATION' and 'EXERCISE') and show a high frequency of appearance, as well as a key behaviour in the calculated co-occurrence networks. Another significant finding is that AKs are mostly accepted in the SSC since there has been high percentage of new terms during 2001–2006, although they have a low survival period. These results support a multidisciplinary perspective within the Sport Sciences field of study and a colonization of the field by rehabilitation according to our AK analysis.

## Introduction

Sport Sciences is a field of study that embraces knowledge produced by research around those social practices such as sport, physical activity, play, game and exercise which is developed from different epistemological and methodological research perspectives. In order to understand the nature of this field, the present paper undertakes a grand overview by analyzing the articles published within the research journals that circulate through Web of Science (WOS), one of the main international data bases. This task is particularly difficult since the scientific production in this and other fields has grown exponentially over the years [1–3], and this is closely linked to the growth of scientific journals. These journals increase in diversity and specialization and multiply the number of articles per year. This inordinate growth has evolved with the advent of the digital age, which has facilitated the reduction of publishing costs associated with the old paper-based system. Researchers have also seen how the amount of information contained in an article has spread with the appearance of supplementary materials. In short, this boost in scientific information is causing researchers to have great problems in knowing the state of the art of a particular topic of study.

Paradoxically, this augment of information is not always an advantage for research groups, since much of the findings are diluted in the large number of bytes that are published [4]. For this reason, research teams are including specialists in the recovery, classification and analysis of information, which improves the efficiency of the scientific documentation search [5]. However, despite the fact that information retrieval systems have become more sophisticated over the last few years, the analysis of scientific literature still requires crafting processes that involve expert reading and interpretation.

Researchers write reviews, systematic reviews, meta-analyses or letters to the editor in order to make the main findings accessible in a given field [6]. These works have been gathered in specialized products in the form of journals or databases (e.g., Cochrane Library). The most successful authors concisely summarize the knowledge they have acquired in their laboratories or the interpretation of the results of the best published articles. With enormous effort and remarkable cognitive abilities, researchers use their experience in this type of article to highlight the strengths and weaknesses in a particular subject of study. It seems that the ability of the human brain to include or discard relevant information is one of the most effective ways to address this type of task [7]. Undoubtedly, this huge effort to successfully manage the information overload deserves the production of papers that usually receive more citations and academic recognition.

However, the highly specialized nature of these review works should be balanced with other works of a more general focus in a certain field of study [8]. Science is increasingly universal and interdisciplinary [9], and yet the specialized literature suffers from the lack of an overview. Cross-sectional knowledge of what is being done in a field might feed the imagination of future researchers, thus leading them to pose hypotheses that could break the barriers imposed by super-specialized knowledge. These grand overviews are even more necessary in fields, such as Sports Sciences, which gathers biological, social and humanities-based studies accompanied by other works of inter and cross-disciplinary character. Therefore, the challenge relies on how we address the analysis of an entire field of study with hundreds of thousands published works.

The increase in data and academic documents has boosted data-mining and text-mining disciplines to manage the huge amount of data. Both disciplines automatically look for patterns that underlie the large masses of information. Although data-mining is a promising alternative, intellectual property protection and the lack of open source data are, to date, two obstacles that seem insurmountable in the short term. Nonetheless, text-mining presents some interesting advantages since what the authors write in their articles is usually expressed in natural language, and most of their information is available in open access (e.g., titles, abstracts, keywords, etc.) [10].

Text-mining refers to the process of extracting useful and non-trivial knowledge from different textual databases using various techniques that are automatically applied to digital environments [11]. Although the text-mining term mainly refers to the analysis of an unstructured text, many of its techniques (e.g. link analysis, reduction dimensionality or clusterization) are also used for structured texts. These techniques are mainly used to analyse content published on the Internet [12–14], although they are also used on scientific documents published in repositories or databases. Bibliometrics, as a discipline that studies the behaviour of scientific publications, also applies these analyses to full texts or parts of the text, such as the title, abstract or keywords [15–17].

Among the different sections stored from a document in scientific databases, Author Keywords (AKs) play a prominent role. The investigations around the AKs are numerous and developed from different approaches, although most of them use the counting terms and co-occurrence networks as the main tools [18–24]. Only a few papers over the years have focused their research interests on the dynamics of keywords [25–27]. Several studies also analysed AKs in the whole field of Sport Sciences [28,29] through the subject matter contained in the documents. However, to the best of our knowledge, no work to date has conducted a global analysis of the issues addressed in the Sport Sciences field.

Against this backdrop, the purpose of this study is threefold. First, to detect the most relevant AKs of the Web of Science (WOS) Sport Sciences Category (SSC) as a representation of the field of study that bears the same name. Second, to discover the dynamics of these words over the years and how these AKs are related to each other, thus giving rise to major research topics. Third, to quantify the most innovative words and how they survive throughout the subsequent years.

## Materials and methods

The authors voluntarily choose AKs, thus performing a succinct exercise of representing the whole text of a document [30]. According to Jones and Jackson, "Keywords are a list of words or phrases that are provided by the author and signify the meaning or main ideas presented in the paper" [31].

AKs have advantages when compared to other sections that are stored in databases (e.g., title, abstract). For instance, their volume of information is easier to manage in storing and analysing than other sections. In fact, AKs consume few bytes of information because there are no connectors between words, which consequently facilitates their storage and handling through computer systems. Obviously, other similar sections like the title, abstract or even the full text contain more information, but the analysis of hundreds of thousands of works supposes a very large volume of text that requires very exclusive computer systems that are accessible to few researchers. The AKs sections do not contain irrelevant information, and everything is ‘edible’. In this sense, AKs do not allow for the manipulation of information by the researchers, thus creating an unbiased position compared with the management of sections with more words. Since there is no possibility of selecting or transforming words, AKs allow to accomplish the positivist science postulate that the observer should not influence the phenomenon under study.

### Author keywords selection

AKs selection criteria include belonging to SSC journals and the Science Citation Index (SCI) in which AKs were published. Eighty-one journals were indexed in this category in 2016. All the AKs published in journals of the SSC were the universe of our study.

**Fig 1:**
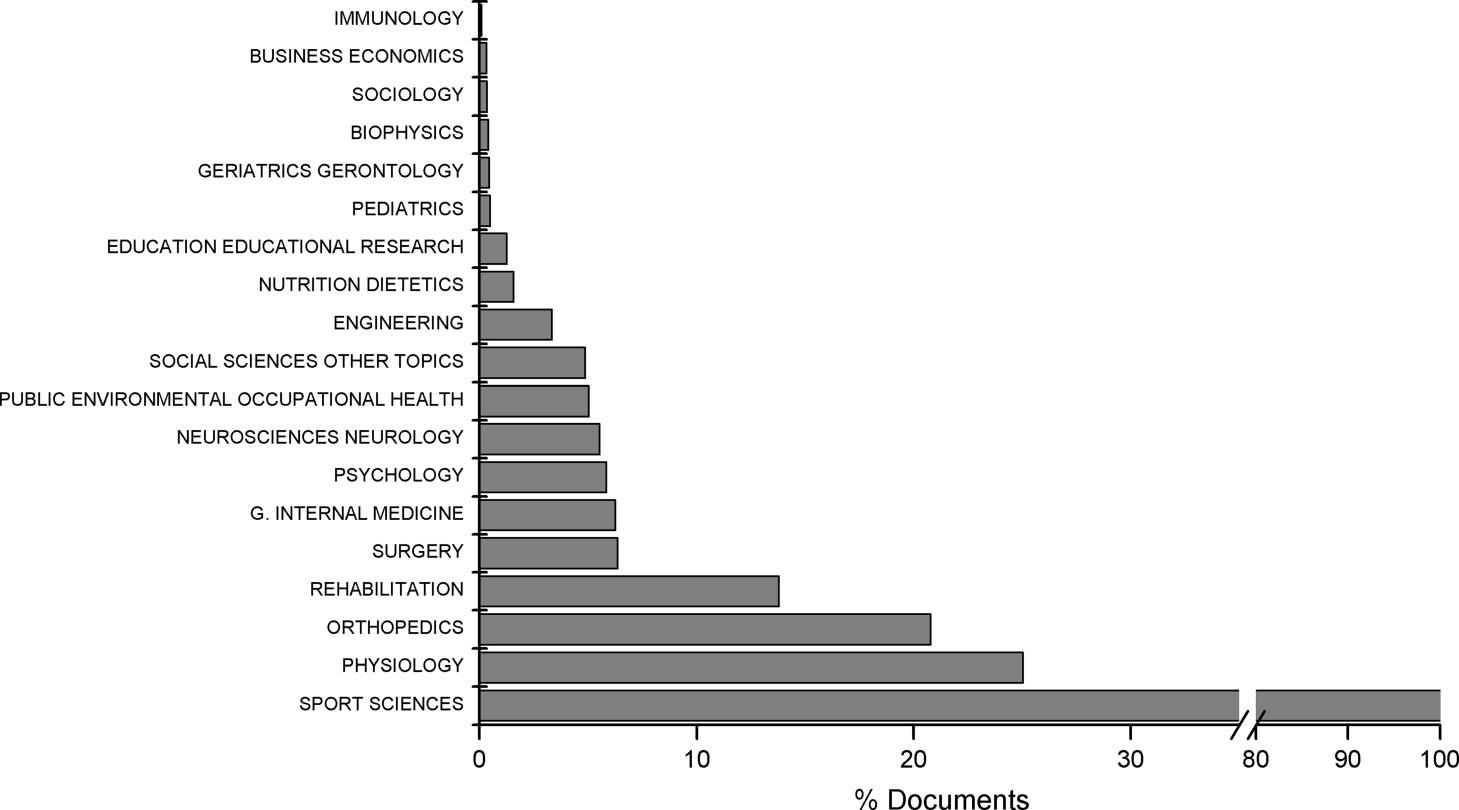
WOS categories from which documents were retrieved.

The SSC ranks 86 out of 234 categories based on the number of journals published. The median Impact Factor of this category is 1.681, which ranks it as the 104th out of 234 categories. Fig 1 shows the WOS categories from which Sport Sciences documents are retrieved. As can be observed, the three categories with the highest representation beyond the specific SSC are (in this order) Physiology, Orthopaedics and Rehabilitation.

The Journal Citation Reports from WOS was used as the tool for searching the journals and the AKs. The ISSNs of each of the journals included in the SSC were downloaded and combined into a single equation. The file used for searching is included in Supplementary Material (S1 File).

The equation was introduced 2017-09-19 in the WOS Core Collection database and it was established the publications before 2017 as the time limit. Only articles, reviews and letters were considered for this analysis. There was no restriction to the language because English is compulsory for title, abstract and keywords. The aforementioned search yielded 168,299 documents. The records were downloaded in batches of 500 documents in plain text format with the following fields: i. Author keywords, ii. Year Published, iii. Subject Category, iv. Publication Name, v. ISSN, vi. Times Cited and vii. Unique Article Identifier. The downloaded files were stored on a hard drive for further analysis.

To obtain and store the AKs in a single document, the software bibexcel (version 2011-02-03, Olle Persson, Umeå University, Umeå, SWE) was used. Since there was no declared AK field in many journals or it was recently declared, the resulting file contained fewer references than the original search. The final document was stored in plain text, and the fields associated with each keyword were separated through a tabulator.

### Author keywords description and co-occurrence analysis

The retrieved AKs were described through frequency tables. The number of times that AKs appeared and their dynamics throughout the period were studied. Those keywords with high frequency appear in the results section, and all of them can be observed in S1 Table. The cleaning process of the 100 most frequent words was done manually, mainly unifying the terms that were in singular and plural and acronyms. For instance, SPORT and SPORTS or ELECTROMYOGRAPHY and EMG.

A citation analysis of the articles in which the AKs had been published was also carried out. With the total number of appointments, the following parameters were calculated: i. Number of citations per article that contained the AK, ii. Percentage of articles that contained the AK that had never been cited, iii. Number of citations obtained by the most cited article that contained the AK, and iv. Hirsch index [32]. Additionally, the three most frequent words that appeared in the journals belonging to the Sport Sciences category were calculated.

A co-occurrence analysis was also performed with the AKs in order to show the number of times that two words appeared simultaneously in a published article. This relationship is established with greater or lesser strength depending on the repetition of this pair of words in a published paper. The co-occurrences of AKs form a graph in which the nodes are the AKs and the edges are the co-occurrence relationships between them. As in any graph, the importance of the nodes can be measured through different parameters of centrality.

We used the bibexcel software to create the AKs’ co-occurrence network, and only the co-occurrences that appeared at least two times or more were taken into account. Pajek software (version 5.01, Batagelj and Mrvar, University of Ljubljana, Ljubljana, Slovenia) was used to visualize and perform the centrality calculations of the network. To facilitate the visual interpretation, several reductions of the original network were made, such as eliminating those edges that had smaller values. The following centrality parameters were calculated: i. All Degree, ii. All Proximity Prestige, iii. Betweenness centrality and iv. Average Distance from All Domain. These are usual parameters to describe the importance each node has within the network. A more detailed description of the equations used for its calculation can be found in previously published works [18,33,34]. In order to locate the nodes in a two-dimensional space a Kamada-Kawai algorithm was used [35]. Once nodes were located, small manual changes were developed to improve the visibility of labels.

The clusters originated from the most important co-occurrence relationships in the last network were also calculated. We used VOSviewer software (version 1.6.4, Nees Jan van Eck and Ludo Waltman, Leiden University, Leiden and Erasmus University, Rotterdam, Netherlands) to establish the clusters, especially the equation that produces the density maps and calculates the resulting clusters [36–38]. This software uses a new mapping technique called VOS, which stands for visualization of similarities. In a simplified way, the equation proposed by the authors calculates the forces of repulsion and attraction of the different nodes as a function of the distance and the strength that joins them. Finally, the number of clusters depends on the resolution that is applied. In our case, this parameter took the value of γ = 1.

An analysis of the clusters dynamic through time was performed using a heat map, in a similar way as it has been done with AKs frequencies. In this analysis the results were expressed as parts per unit regarding to the highest count of a specific AK within a cluster during a year (highest value = 1).

### New author keywords search from 2001–2006

We choose a six-year period (2001–2006) as a first step to locate new AKs, since there were no previous studies that use this type of methodology. This period is in between of many previous years and sufficient subsequent years with AKs to conduct the analysis of new words with guarantees.

In order to locate the new words and to track their frequencies of appearance during the 10 subsequent years, custom-written software routines were established (MATLAB R2013a, MathWorks Inc., Natick, MA, USA). Any word variation from previous ones was considered as a ‘new’ word and included those that were written with some orthographic or typographical mistakes. However, the ‘mistaken’ words will likely disappear over the years, thus becoming anomalies without impacts. If anomalies still survive, then it would be a case of new use accepted by the scientific community, and consequently, it would have an impact on the scientific writings. We only made two exceptions to this rule: i) differences between uppercase and lowercase were not considered, and ii) Hyphens were removed. For instance, Meta-analysis → Meta analysis.

As a necessary step prior to the analysis, the records were divided into three periods based on the years in which the AKs were published: 1) the historical period [1889–2001], 2) the onset period [2001–2006], and c) the survival period [10 subsequent years 2002–2011, 2003–2012 (…) 2007–2016]. Once the periods were established, a retrospective search was started with the words published in the period of appearance looking back into the historical period. Those words that had never been published in the historical period were selected as new words in the SSC.

Later, a search was made in the survival period with the new words selected. The search was conducted year after year, thus resulting in a vector of 10 columns for each word in which 1 indicated the appearance of the term and 0 indicated no appearance. In addition, the number of times the word appeared every year was counted. Frequency tables were calculated for the entire generated file.

### New author keywords analysis during the survival period

With the new words detected, a first analysis was carried out. It consisted of calculating the probability that each new word had to survive or disappear. An analysis of the survival through Kaplan-Meier curves was then proposed. This type of analysis estimates the time that passes until a certain event occurs. This analysis can be used beyond the estimation time until death since the survival analysis can be applied to all those events that occur over time and have been previously defined. In fact, this type of analysis has been applied to a large number of fields of study, such as medicine, economics, production engineering and social sciences [39].

Before conducting this analysis, it is necessary to establish three fundamental aspects: the time of observation, the moment in which the event of interest occurs and when a subject is censored. In our analysis, the research subjects are the AKs, and they were followed up over the survival period. That is, we are going to test over 10 years if these words appear in an article published in the SSC.

Regarding the definition of the event, it is necessary to consider that a word can appear and disappear discontinuously over a period of 10 years. Therefore, it could be argued that the data is interval-censored [40]. However, we think that as long as there are subsequent records in which the word appears, a particular AK is alive as researchers use it while preparing their articles. As a general criterion, it was established that a word ‘died’ in the following year of its last appearance in which there were records. When this circumstance occurred, this year was indicated as the moment in which the event occurred and then the analysis stopped for this word [39].

The last step in our analysis was to establish which words were censored. In our work, only those words that appeared throughout the observation period were considered censored (right-censoring).

Once the data matrix was prepared, the analysis was performed using the SPSS 20 software (IBM, Armonk, USA). We calculated the Kaplan-Meier curves for the total set of new words, the average survival times, and the 95% confidence intervals.

## Results

### Descriptive statistics

**Fig 2:**
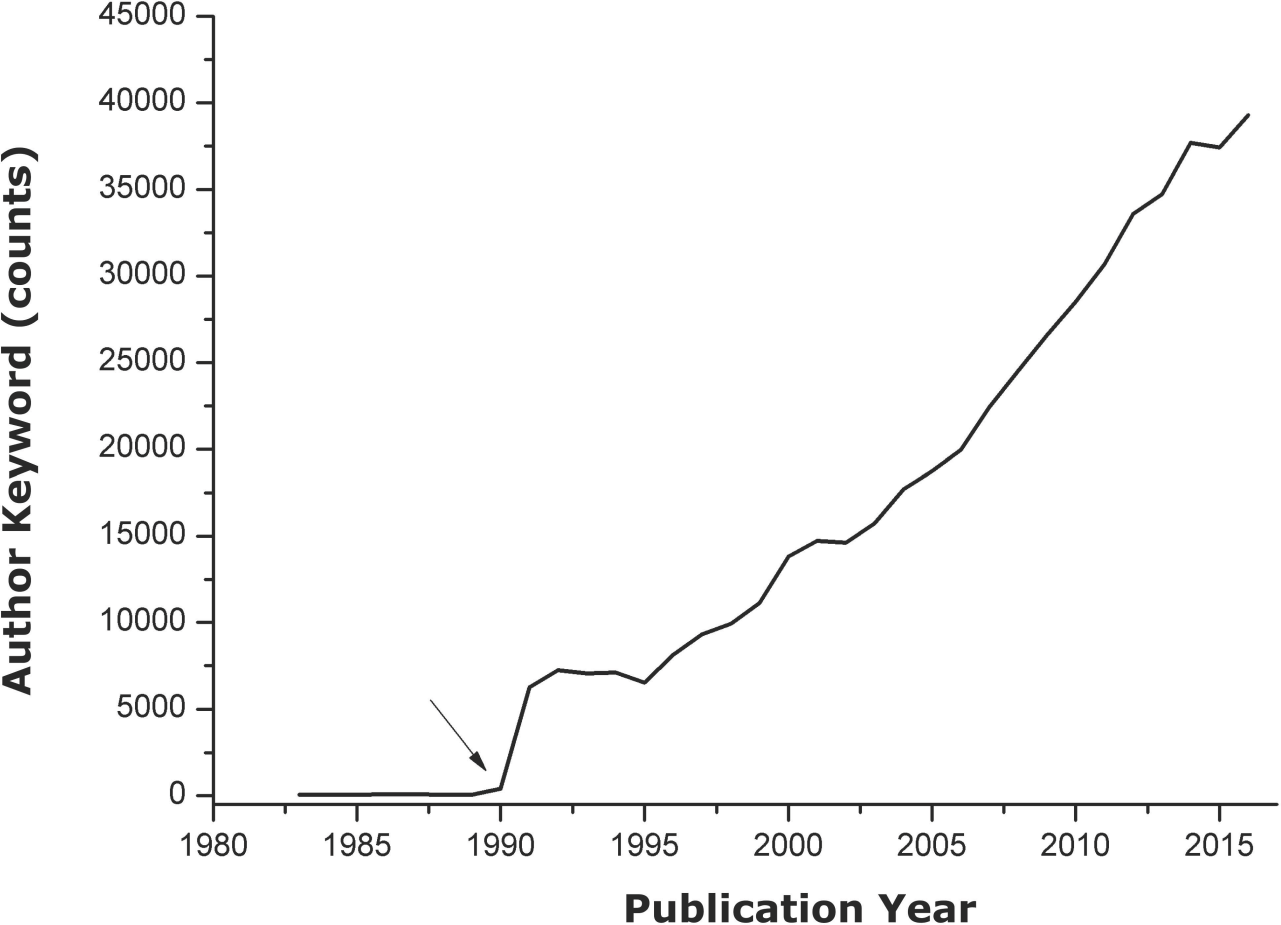
Frequency of AK appearance in sport sciences category throughout the 1983–2016 period. The arrow indicates that 1991 was when AK usage began to be generalized.

In all, 111,606 documents that contained AKs were obtained from a total of 168,299 records. The first AK of the SSC appeared in 1983. As expected, the amount of AKs has been growing over the years due to the increase in scientific production and the popularization of AKs usage in the field for indexing papers. AKs usage was not generalized until 1991, as can be observed in Fig 2. A total quantity of 504,479 AKs were recorded between 1983 and 2016. This amount is reduced to 101,824 AKs when duplicate words are discarded. Although there is an average of four AKs per article, 27 AKs were observed in a particular paper [41].

**Fig 3:**
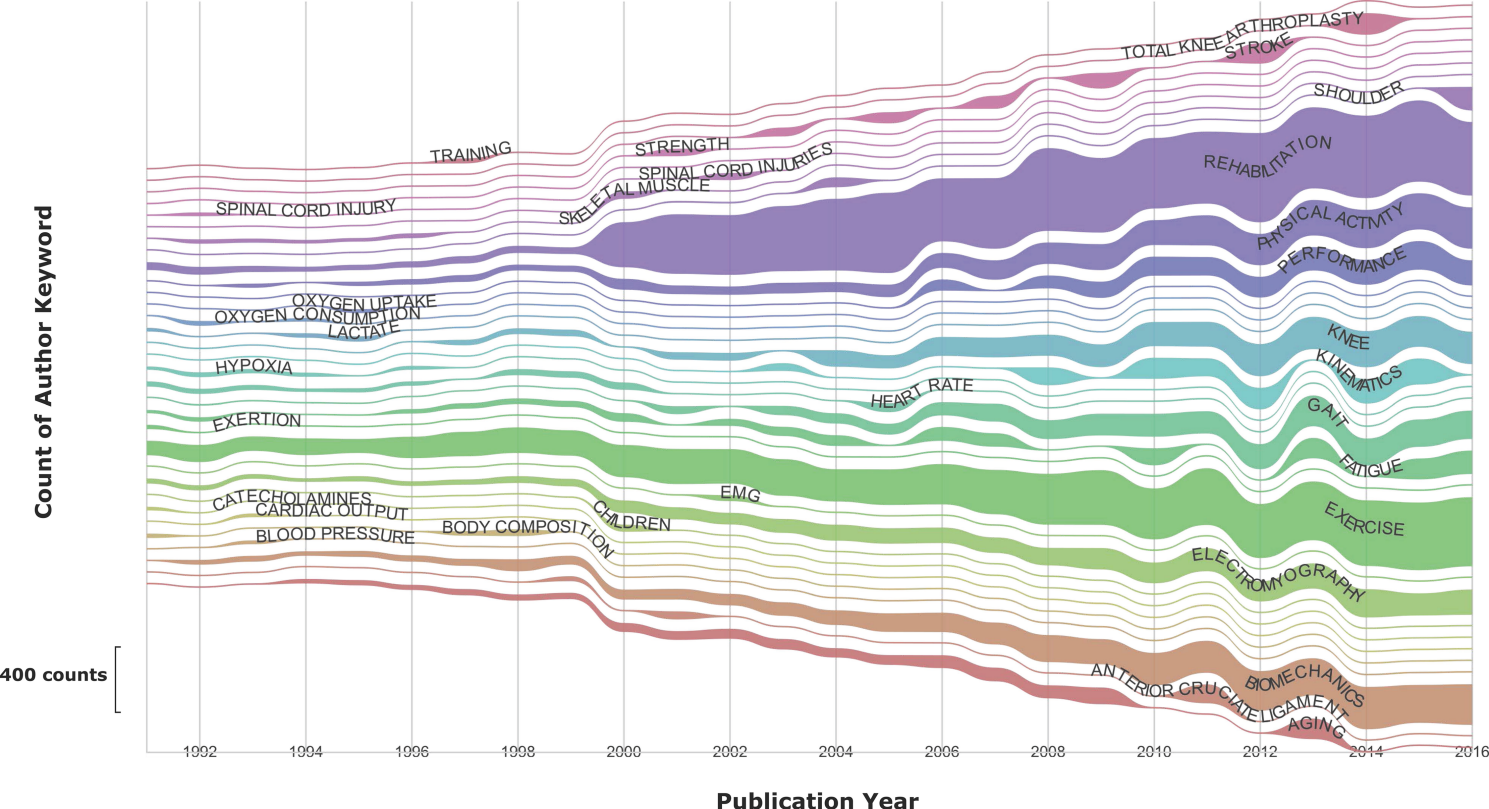
Changes in the frequency of the 10 most frequent AKs over the years from 1991–2016. No AKs before 1991 appear in the figure because the total frequencies are under 30 AKs. A total of 31 AKs among the top frequent ten were identified during the analysed years. The size of the lines is proportional to the count of the number of times they appear in a given year. In the first year (1991), the total frequency was 363 AKs, and in the last year the total frequency of the 10 most used words was 3294 AKs.

Twenty-six words appeared more than 1000 times over the tested years (Table 1). The most repeated word was 'REHABILITATION' followed by 'EXERCISE'. Both terms represent approximately 1.5% of the total. Words with a lower frequency of appearance can be found in S1 Table. Regarding the evolution of these words over the years analysed, Fig 3 shows how 'EXERCISE' was the most used AK until 1999, and 'REHABILITATION' became the predominant AK in the following years.

**Table 1:** Author keywords that appeared more than 1000 times during the 1983–2016 period in the sport sciences category.

Author Keyword	Counts
REHABILITATION	7647
EXERCISE	5975
ELECTROMYOGRAPHY	3136
BIOMECHANICS	2961
PHYSICAL ACTIVITY	2510
KNEE	2273
ANTERIOR CRUCIATE LIGAMENT	2199
GAIT	1996
FATIGUE	1700
AGING	1671
PERFORMANCE	1622
KINEMATICS	1610
SPORT	1519
SHOULDER	1394
OXYGEN CONSUMPTION	1353
STROKE	1316
ATHLETES	1313
STRENGTH	1253
SPINAL CORD INJURIES	1235
HEART RATE	1223
TRAINING	1214
INJURY	1186
MAGNETIC RESONANCE IMAGING	1180
CHILDREN	1119
SOCCER	1050
RUNNING	1007

The AKs had an average number of 19 characters in length. Particularly, 0.4% of the AKs had a greater length than 50 characters, and only 5 AKs had a length of 1 character.

Regarding citations received by articles which contain the main AKs, Table 2 shows how the words ‘AGING’ and ‘PHYSICAL ACTIVITY’ were more efficient. On the contrary, ‘SPORT’ and ‘ATHLETES’ were the less efficient words.

**Table 2:** Citation parameters for articles which contain the most popular AKs in the sport sciences category.

Author Keyword	citations / artícle	% artícles without citations	h-index	Maximum number of citation of an article	Unique Article Identifier*
AGING	31.55	5.45	101	1111	WOS:000227665400002
PHYSICAL ACTIVITY	29.01	9.04	115	2417	WOS:A1993KG53700011
EXERCISE	27.56	8	146	3185	WOS:000089257400009
CHILDREN	25.79	7.77	75	805	WOS:000207606400001
GAIT	25.72	5.91	96	979	WOS:000177649600002
ANTERIOR CRUCIATE LIGAMENT	24.96	6.91	89	362	WOS:000169666000011
STROKE	24.47	4.94	80	567	WOS:000231077900028
FATIGUE	24.32	6.88	86	455	WOS:A1996VA58600029
REHABILITATION	24.06	5.57	133	921	WOS:000170275000007
OXYGEN CONSUMPTION	23.66	6.95	75	1185	WOS:000292773000025
STRENGTH	23.56	8.06	79	589	WOS:000185850300013
MAGNETIC RESONANCE IMAGING	23.31	5.34	64	703	WOS:000088230400011
INJURY	22.89	9.19	74	369	WOS:000072475800012
RUNNING	22.75	9.04	71	649	WOS:000084834900012
SPINAL CORD INJURIES	22.66	3.97	60	517	WOS:A1992HX73900002
HEART RATE	21.95	9.4	72	565	WOS:000167227800019
ELECTROMYOGRAPHY	21.83	7.08	87	603	WOS:000178034600020
KINEMATICS	21.5	8.7	83	369	WOS:000072475800012
BIOMECHANICS	21.18	6.35	98	555	WOS:A1992JX14100009
SOCCER	21.11	12.76	72	446	WOS:000230141300007
KNEE	20.94	7.22	92	362	WOS:000169666000011
TRAINING	20.57	8.98	70	423	WOS:A1995RV19200001
PERFORMANCE	18.91	8.2	76	431	WOS:000263752200026
SHOULDER	18.39	8.32	70	335	WOS:000083353800002
SPORT	15.41	15.14	66	391	WOS:A1997XJ67700017
ATHLETES	14.42	14.85	58	289	WOS:000230326800005

Data are ordered according to the number of citations by article which contains the AK. h-index = Hirsch index.

* Indicates the WOS identifier of the most cited paper (for searching purposes, include this identifier in the Advanced Search tag field UT).

It can be observed in Table 3 the AKs most used within the journals with top ten impact factor in 2016 in SCC.

**Table 3:** Most used author keywords within the top ten journals of the sport sciences category.

Journal*	AK 1	AK 2	AK 3
Exercise Immunology Review	EXERCISE	INFLAMMATION	CYTOKINES
British Journal of Sports Medicine	EXERCISE	INJURY PREVENTION	EPIDEMIOLOGY
American Journal of Sports Medicine	KNEE	ANTERIOR CRUCIATE LIGAMENT	SHOULDER
Exercise and Sport Sciences Reviews	EXERCISE	AGING	SKELETAL MUSCLE
Medicine and Science in Sports and Exercise	EXERCISE	PHYSICAL ACTIVITY	AGING
Journal of Science and Medicine in Sport	PHYSICAL ACTIVITY	EXERCISE	CHILDREN
Journal of Applied Physiology	EXERCISE	SKELETAL MUSCLE	HYPOXIA
Scandinavian Journal of Medicine & Science in Sports	EXERCISE	PHYSICAL ACTIVITY	SOCCER
Archives of Physical Medicine and Rehabilitation	REHABILITATION	SPINAL CORD INJURIES	STROKE
Knee Surgery Sports Traumatology Arthroscopy	KNEE	ANTERIOR CRUCIATE LIGAMENT	TOTAL KNEE ARTHROPLASTY

*Sports Medicine is not included in the table because this journal does not accept AKs. AK1, AK2, AK3 the three more frequent words in each journal, ordered from major to minor.

### Author keyword co-occurrence and clusters

**Fig 4:**
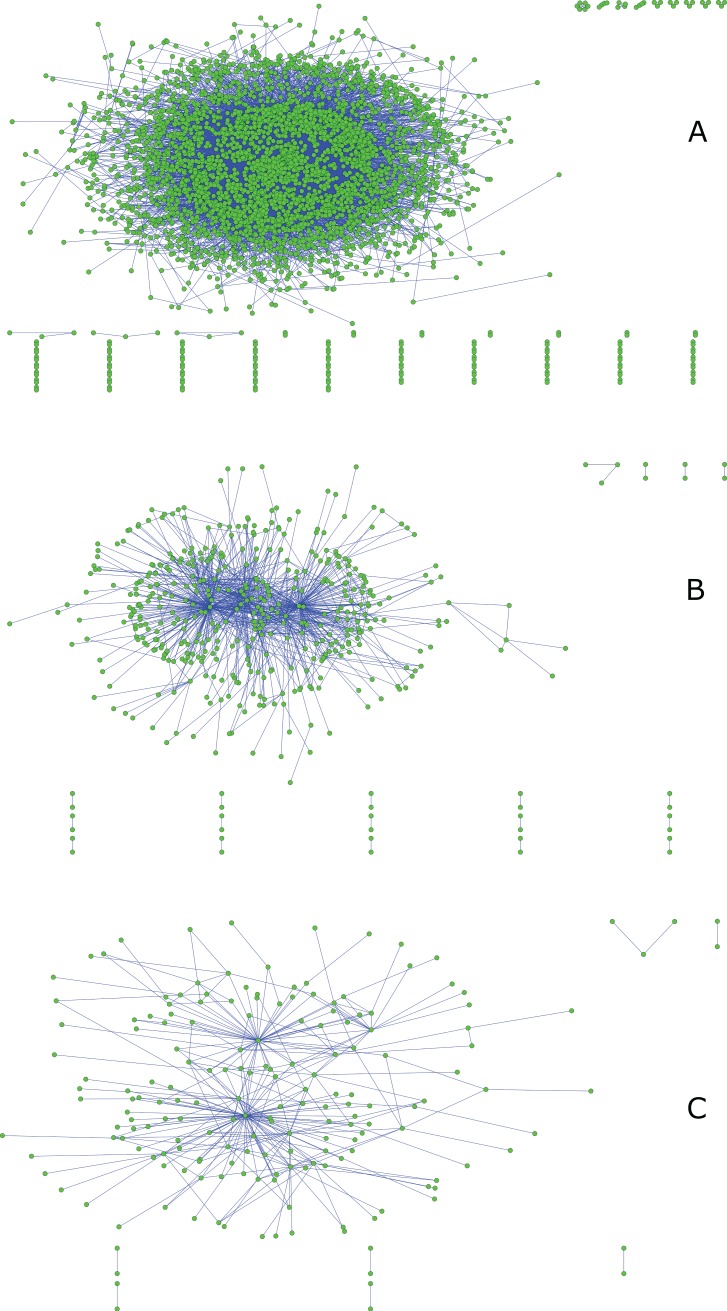
Author keyword networks co-occurrence. (A) shows the network of co-occurrences with 3994 nodes (threshold≥ 5). (B) shows the reduction of the network (threshold≥25) with a total of 469 nodes. (C) shows 185 nodes with the highest co-occurrence values (threshold≥50).

A co-occurrence network was built with a total of 504,479 AKs. Only those words that at least appeared accompanied by others a minimum of 2 times were considered for elaborating the co-occurrence matrix. The resulting network had 101,757 nodes and 729,800 edges. To visually represent it with a manageable number of words (nodes), several reductions were made. The process of reducing the general network is graphically represented in Fig 4.

Fig 4A shows the first reduction (threshold*≥* 5). It is a network with 3994 nodes and a total of 15037 edges. The average degree of the network is 7.52. The largest distance between nodes was 10 jumps between the words 'BREATH HOLD' and 'SENSORIMOTOR SYNCHRONIZATION'. In Fig 4B the edges with values less than 25 (threshold*≥* 25) have been eliminated and a network with 469 nodes was presented. Finally, a more drastic reduction is presented in Fig 4C. In this graph, only those edges with values above 50 co-occurrences (threshold*≥* 50) appear. It is then possible to visualize a network with 185 nodes and 302 lines. This network visually facilitates its interpretation, but the number of nodes is still very large. Therefore, a network only with co-occurrences greater than 100 was used for centrality calculations and cluster extraction. All networks centrality parameters can be observed in S2 Table.

Table 4 shows the centrality values of the 20 most prestigious AKs within the network. The AK that reached the greatest number of co-occurrences was 'REHABILITATION', with it concurrently being the word with the highest degree and betweenness. This AK was also the one that had the lowest average distance (1.44 jumps) from the other AKs. The remaining values for the set of nodes can be observed in S2 Table.

**Table 4:** Centrality parameters of author keywords co-occurrence network.

Author Keyword	All Degree	All Proximity Prestige	Betweenness centrality	Average Distance from All Domain
REHABILITATION	39	0.616	0.662	1.446
EXERCISE	15	0.459	0.283	1.938
BIOMECHANICS	8	0.416	0.027	2.138
AGING	3	0.416	0.024	2.138
MUSCLE	3	0.416	0.005	2.138
KNEE	9	0.405	0.089	2.200
GAIT	10	0.402	0.044	2.215
ELECTROMYOGRAPHY	7	0.394	0.042	2.262
ANTERIOR CRUCIATE LIGAMENT	4	0.391	0.024	2.277
SHOULDER	5	0.388	0.058	2.292
OSTEOARTHRITIS	2	0.381	0.000	2.338
BALANCE	3	0.378	0.001	2.354
STROKE	2	0.376	0.000	2.369
WALKING	2	0.376	0.000	2.369
CEREBRAL PALSY	2	0.376	0.000	2.369
POSTURE	2	0.369	0.000	2.415
SPINAL CORD INJURIES	1	0.366	0.000	2.431
BRAIN INJURIES	1	0.366	0.000	2.431
OUTCOME ASSESSMENT (HEALTH CARE)	1	0.366	0.000	2.431
QUALITY OF LIFE	1	0.366	0.000	2.431

The table shows the 20 words with the highest Proximity Prestige value. The parameters have been calculated over a network with 74 nodes (threshold≥100)

**Fig 5:**
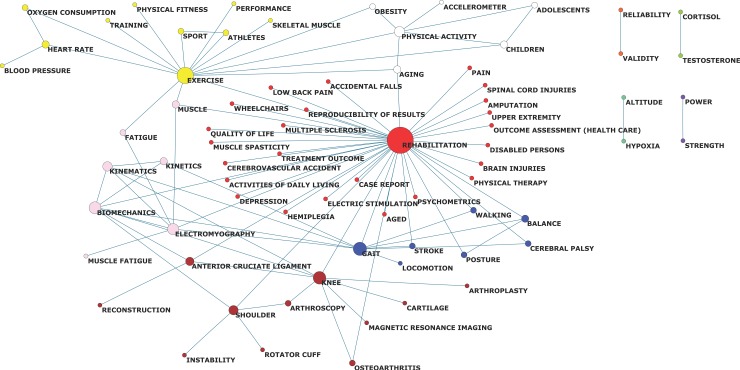
Main research topics in the sport sciences category according to the co-occurrence of author keywords. 6 large clusters appear with different colours as follows: Red = Rehabilitation, Yellow = Exercise and training, Pink = Biomechanics, Garnet = traumatology, Blue = Gait and balance, and White = Physical activity. The size of the nodes indicates co-occurrence between related terms, and a larger size means a greater co-occurrence.

In a second level of analysis, we grouped the AKs into clusters in order to indicate which topics are the most common in the SSC. The clusters are represented with different colours in Fig 5. In our analysis, there are 6 major themes that are related to each other.

The most central cluster of all is commanded by the word 'REHABILITATION' and appears in red. Several medical terms about musculoskeletal and nervous system pathology share a space in this cluster with different physiotherapy techniques.

The cluster commanded by the word 'EXERCISE' appears in yellow, and it exerts absolute control over other terms. It is a cluster in which training and physiology have an important position.

In pink and blue are two clusters close to each other. The first refers to the 'BIOMECHANICS' and the second to postural control. In between biomechanics and postural control, a cluster whose main theme is traumatology is coloured in garnet.

A sixth cluster is formed by the words 'PHYSICAL ACTIVITY' and is related to different moments of the life cycle.

**Fig 6:**
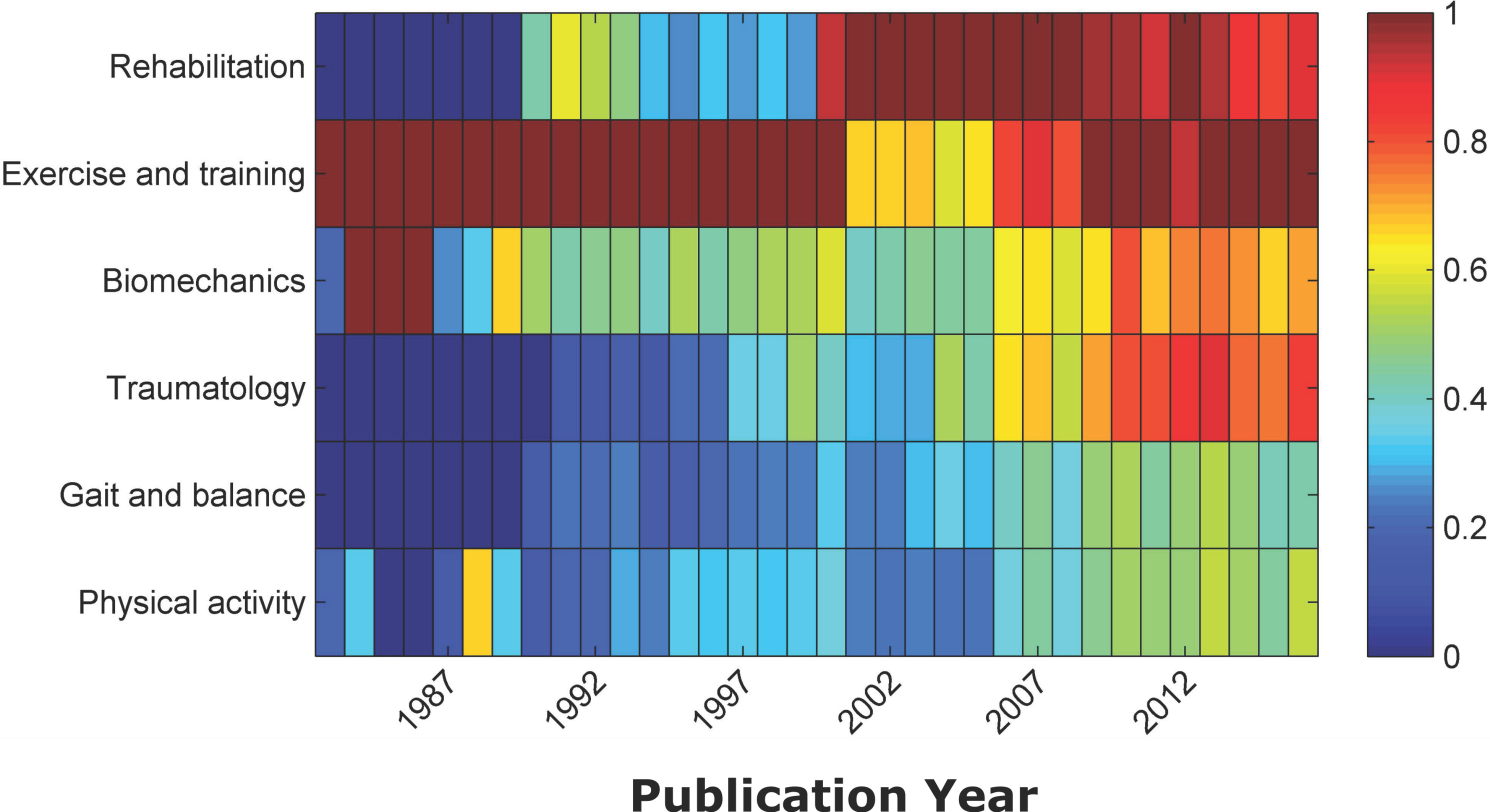
Heat map on the AKs dynamic contained in each cluster. Data were expressed as parts per unit regarding to the highest count of a specific AK within a cluster during a year (highest value = 1).

It can be seen in Fig 6 the dynamics of each cluster along the years. The ‘EXERCISE AND TRAINING’ cluster ‘was the predominant at the end of the past century, given this place to ‘REHABILITACION’ later on.

### New author keywords appeared in the period 2001–2006 and survival in the following years

During the years from 2001–2006, a total of 21,662 new AKs were published, which corresponds to 42.31% of the total AKs published in this period. Among the new ones that appeared in this period, those that had greater success during the following years were 'POSTURAL BALANCE', 'DOUBLE BUNDLE', 'PERFORMANCE ANALYSIS' and 'COMBAT SPORTS', which had total frequencies above 100 (Table 5). The word that had a stronger debut was 'PHYSICAL THERAPY TECHNIQUES', which was used for the first time 11 times in 2003.

**Table 5:** New author keywords in 2001–2006 period and frequency of use during the following 10 years.

New Keyword	Freq.	New Keyword	Freq.	New Keyword	Freq.	New Keyword	Freq.	New Keyword	Freq.	New Keyword	Freq.
2001	(2002–2011)	2002	(2003–2012)	2003	(2004–2013)	2004	(2005–2014)	2005	(2006–2015)	2006	(2007–2016)
PARKINSON DISEASE	46	ICF	89	TRAINING LOAD	46	POSTURAL BALANCE	158	DOUBLE BUNDLE	126	OSTEOCHONDRAL LESION	46
FEMOROACETABULAR IMPINGEMENT	34	AUTOLOGOUS CHONDROCYTE IMPLANTATION	57	CAREGIVERS	31	LOCKING PLATE	57	PERFORMANCE ANALYSIS	123	AUTONOMY SUPPORT	45
EVIDENCE BASED MEDICINE	32	TYPE 2 DIABETES	57	REVIEW (PUBLICATION TYPE)	31	REPEATED SPRINT ABILITY	49	COMBAT SPORTS	108	SEDENTARY LIFESTYLE	44
MUSCLE, SKELETAL, PHYSIOLOGY	32	POSTACTIVATION POTENTIATION	42	MEDIATION	30	ANTERIOR CRUCIATE LIGAMENT (ACL) RECONSTRUCTION	46	TALENT DEVELOPMENT	50	TROCHLEAR DYSPLASIA	41
RANDOMIZED CONTROLLED TRIALS	31	NIRS	33	PHYSICAL THERAPY TECHNIQUES	30	CORE STABILITY	44	TIBIAL SLOPE	42	AMPK	39
SPORTS NUTRITION	28	AKT	28	HANDWRITING	29	CONCUSSIONS	36	GLOBAL POSITIONING SYSTEM	36	RELATIVE AGE EFFECT	39
ACHILLES	27	GLUTEUS MEDIUS	28	MOSAICPLASTY	28	ADIPONECTIN	33	SMALL SIDED GAMES	34	PGC 1 ALPHA	38
SHOULDER ARTHROPLASTY	27	SUBSCAPULARIS	24	ACTIGRAPH	24	POLICY	33	MYOSTATIN	31	ORTHOPAEDIC TRAUMA	37
QUALITATIVE	26	OVERHEAD ATHLETE	21	BIOMARKERS	23	SLAP	30	VALIDATION STUDIES	29	INERTIAL SENSORS	36
AUSTRALIAN FOOTBALL	24	MINIMALLY INVASIVE SURGERY	20	DOUBLE BUNDLE RECONSTRUCTION	23	HIGH INTENSITY INTERVAL TRAINING	29	COMBAT SPORT	23	ANATOMIC RECONSTRUCTION	34
HAMSTRING TENDON	24	WHIPLASH INJURIES	20	FALL PREVENTION	21	PACING STRATEGY	29	INCLUSION	22	MESENCHYMAL STEM CELLS	31
STEADINESS	24	COMPUTER NAVIGATION	19	NEURONAL PLASTICITY	21	INTRA ARTICULAR	28	SURFING	20	MTOR	30
MALUNION	23	BALL SPEED	18	MULTIFIDUS	20	GAIT VARIABILITY	27	AUTOLOGOUS CHONDROCYTE IMPLANTATION (ACI)	19	SPORTS INJURY PREVENTION	30
PROSTHESES AND IMPLANTS	23	SUTURE	18	NEUROMUSCULAR TRAINING	20	POST ACTIVATION POTENTIATION	27	TROCHLEOPLASTY	19	TIME MOTION	30
SICK LEAVE	23	ARTHROSCOPIC SURGERY	17	SEMG	20	MICROARRAY	25	BUILT ENVIRONMENT	18	SUBCHONDRAL BONE	27
CENTRAL ACTIVATION	19	MMG	17	GHRELIN	18	COLLISION SPORT	24	MPFL RECONSTRUCTION	18	OSTEOCHONDRAL ALLOGRAFT	26
CHONDROCYTES	19	MUSCULOSKELETAL DISEASES	17	PATELLOFEMORAL INSTABILITY	18	IDENTITY	24	MESENCHYMAL STEM CELL	17	LOCKED PLATING	24
ENDOTHELIAL DYSFUNCTION	19	PERFORMANCE INDICATORS	17	TRAINING STATUS	18	CORE	23	REVISION TOTAL KNEE ARTHROPLASTY	17	VIBRATION TRAINING	24
AUGMENTATION	18	OLDER PEOPLE	16	VIBRATION EXERCISE	18	GAME ANALYSIS	22	SELF CONTROL	17	POST EXERCISE HYPOTENSION	23
HEART RATE RECOVERY	18	ELITE SPORT	15	PHYSICAL EDUCATION TEACHER EDUCATION	17	SKELETALLY IMMATURE	22	SPORT PHYSIOLOGY	17	POSTEROLATERAL BUNDLE	23

The Author Keywords are ordered from the highest to the lowest frequency. The table only shows the 20 most used Author Keywords for each respective year.

Only 61 journals accepted new AKs during the 2001–2006 period out of the total number of journals in the SSC. The Journal of Applied Physiology, Medicine and Science in Sports and Exercise and Aviation Space and Environmental Medicine were the ones that published a greater number of new AKs among all the analysed ones.

**Fig 7:**
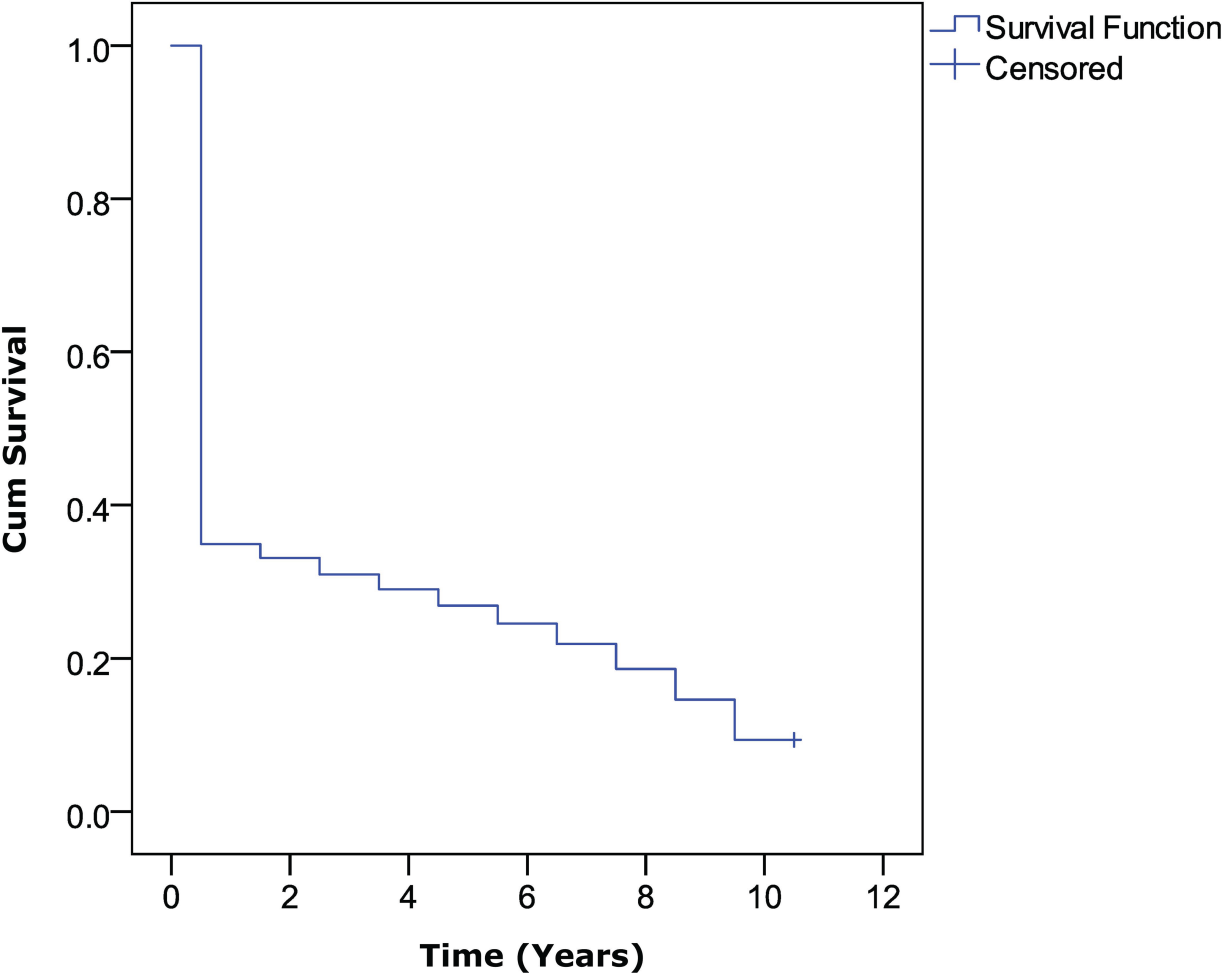
Kaplan–Meier curves of the ten years following of a new AK.

Fig 7 shows the survival curves of AKs over the 10 years after their appearance. It can be observed that during the first year, more than half of the words disappeared and were not used again until the end of the analysed period. Since then, a soft fall is observed that is accentuated slightly towards the end. Only 9.4% of the words arrived at the end of the period without any event being observed. Moreover, only 2027 words were used during the ten years after its appearance. The average time (95% CI) of survival for the series was 2.93 (2.88 to 2.98) years.

## Discussion

This is the first article in performing an empirical global analysis of the Sport Sciences research, by using AKs from articles contained in WOS, to identify major research trends in this field of study. Previously, only partial and regional analyses had been developed, [28,29,42] but global analyses are necessary for understanding holistically this complex field.

A particular surprising result is the emergence of 'REHABILITATION' as the most frequent AK in the SSC. It is paradoxical that the most common term in this subject category is the one that gives name to another subject category precisely called *Rehabilitation*. According to WOS, the SSC would encompass the following topics:

“Sport Sciences covers resources on the applied physiology of human performance, physical conditioning for sports participation, optimal nutrition for sports performance, and the prevention and treatment of sports-related injuries and diseases. This category also includes resources on sport psychology and sociology”.

These topics make it possible that rehabilitation has a place in this category as part of the treatment of injuries produced during sport practice. However, such a large number of words in SSC requires a more detailed reflection.

Since journals may be assigned to more than one WOS category, 13% of our analysed documents have been published in journals that belong to Rehabilitation and SSC. This percentage is not enough to explain the predominant position of Rehabilitation AK. Since the second category of WOS that shares the most number of documents related to Sports Sciences is Physiology, it was more likely that any word related to this category would have obtained the first positions. Moreover, in one of the few studies analysing the AKs that appeared in one of the most important congresses on sports sciences held in Europe [29], the authors observed a relatively low weight of rehabilitation compared to physiology (2% vs 22%, respectively). This dissonance between our results and those described in the cited study may be due to the European College of Sport Science congress organization and its journal that consciously controls the main themes under which authors present their communications. However, the WOS category is made up of different journals, which have different editors that make decisions in competition with other journals, and consequently, their management is characterized by the decentralization of decisions. Although our data does not allow us to infer why rehabilitation has been imposed in SSC, it seems that journals editors themselves have prioritized the contents related to the secondary prevention of sports injuries.

The second most frequent AK is 'EXERCISE', a key term that undoubtedly is a fundamental part of the SSC. In essence, the word expresses the orderly development of physical activity for the purpose of maintaining or improving physical fitness [43]. Although this word is usually associated with training (as will be seen later in this discussion), its use permeates much of what is published in the area [28]. However, the absence of some terms that should theoretically emerge among the most cited in the SSC is astonishing. For instance, the term 'SPORT' is not among the most repeated words, as would be expected since it names the category. However, the term could be obliterated because the authors may assume it is not necessary to explicitly indicate the word of the category in their publications. Despite this explanation, it is still surprising that among the most cited words there is only one sport, 'SOCCER'.

The academic sport disinterest shown by our results has already been previously proven by Stone et al. in 2004 [44]. According to these authors, sport as a subject matter is being displaced by others more focused on biomedical aspects related to the practice of exercise. They argue for the methodological difficulties (i.e., internal validity vs. external validity), little training of the coaches who are consumers of the final product, deficient training of university students about the sport and scarce employability of sport scientists. Although our results do not allow us to know why researchers choose a certain subject of study, we think that the system of academic rewards are influencing the decision-making of researchers about their topics of study. This idea is reinforced by the statistics obtained regarding the citation parameters, where the word ‘SPORT’ gets the worst results.

Researchers who publish in journals with a high impact factor are more likely to obtain relevant positions within academic institutions and more funding for their projects [45,46]. Researchers choose topics that are of interest to the journals with the greatest impact, and they are inclined towards topics that are more likely to be cited, thus entering a vicious circle from which it is difficult to leave. The journals are also part of this ‘game’ and are not exempt from pressure. Since prestige is associated with journal citations, it is possible that the best placed journals tend not to accept breakthrough ideas because they increase the quotations with mainstream themes that allow them to preserve their prestige. It is possible that, since the biomedical sciences have a long research tradition and well-structured methods, the topics associated with the word 'SPORT' may not be too attractive for journals. However, our experimental data does not allow us to conclude in this direction. We believe that future work should address this problem by looking at other factors or co-variables that explain the phenomenon.

Regarding the dynamics of the AKs, the last 20 years are characterized by showing few changes with respect to the analysis of the total frequency. It indicates that the SSC is quite stable over time. Both words, 'REHABILITATION' and 'EXERCISE', have a hegemonic position since AKs were introduced in the normalization of SSC journals. There are no themes that appear and then disappear. Perhaps the only exceptions to this rule are the 'AGING', 'STROKE' and 'TOTAL KNEE ARTHROPLASTIA' AKs that during the years 2012–2014 show a slight increase in their frequency of appearance. These words are directly or indirectly associated with the ageing process. Therefore, their appearance among the most cited words in the last decade may be strengthened, due to the growing concern with the progressive ageing of the population in developed countries.

An analysis of AKs alone does not offer an accurate view of what occurs in a field of study, since AKs only express a part of the articles content and are usually used as a claim for readers. Therefore, a more complex analysis focused on the connections among words (such as their co-occurrence) may enrich the view of the field. In fact, our analysis of co-occurrences among AKs has yielded interesting results. When authors match two terms in a single article, they are indicating the use of different topics to solve a particular problem. In our co-occurrence networks, it can be seen that the words 'REHABILITATION', 'EXERCISE' and 'BIOMECHANICS' are the ones that obtain the highest values in the centrality parameters that were analysed. It is especially revealing that the betweenness value obtained reflects the mediating role that 'KNEE' plays as a gateway for traumatology in our graph. Beyond the individual values that each AK obtains, the co-occurrence analysis ultimate aim is to obtain clusters that trace the predominant themes.

The clusters obtained in the results show that the rehabilitation and biomechanics AKs (two names of recognized disciplines) come together with physiology and traumatology within the SSC. It is an amalgam of multiple AKs that are fundamentally related with biomedical disciplines, while words from other social and human sciences are absent. If the SSC is intended to represent the Sport Sciences field of study in the WOS, it should expand the articles from the social and human sciences. The editors of the journals and WOS managers should assume this task, since the editors are members of the Sport Sciences community with responsibilities within the field of study [47,48], and the WOS managers are accountable for a balanced selection of journals from the field of study as a whole.

However, authors of the scientific community make the decision to write the AKs in their articles, and thus they are also responsible for the disciplinary and thematic mixtures reflected in the clusters. These clusters reflect a field of study that has not achieved an international consensus to define itself as a scientific discipline with a clear subject matter, as various authors have noted [49–51]. Since Henry [52] opened up this issue in the mid-1960s, various disciplinary proposals [53–55] and several contributions around its subject matter [56–59] have been made. These proposals have been unevenly followed, and a multidisciplinary perspective seems to have been imposed. The interdisciplinary and cross-disciplinary proposals that involve a greater integration of knowledge from biomedical, human and social disciplines present conceptual and practical problems that make them difficult to materialize [49,55,60–62].

Our analysis of AKs supports this multidisciplinary perspective that the Sport Sciences field of study has experienced since the middle of the last century. As Henry stated [63] more than fifty years ago, this field still displays a few common interests, key issues and conceptual systems, and it is not characterized by a single body of knowledge. The Sports Sciences field has been developed as an amalgam of isolated sub-disciplines that are derived from the mother ones and seek rapid academic respectability [64]. Therefore, there are few or no connections among sub-disciplines to build common aspects from all of them. This is indicated by the empirical analysis of the AKs conducted in this work and which is closer to the reflections of authors that refer to chaos [50], fragmentation and over-specialization [49,65] or the lack of integration [63] in this field of knowledge, despite some recent integrative advances [66,67]. In addition, AK analysis reflects a lack of agreement, even in the subject matter that revolves around historically consolidated concepts in the field of study, since terms such as sport, game, body and movement are missing in our analysis. However, the situation of colonization is even worse when there is a predominance of 'REHABILITATION' over other concepts, thus endangering the identity of the field of study by dissolving and absorbing the key concepts of another field of close study that has attracted the scientific interest of our community of researchers. Only 'EXERCISE' and 'PHYSICAL ACTIVITY' have a presence in the cluster analysis. This makes us think that, in addition to epistemological, methodological and conceptual problems, there are practical problems linked to prestige, recognition, employability and ultimately personal survival within academia and science in general.

Finally, the survival analysis of AKs during the years 2002–2007 allows us to establish the quantity of new terms that appear in the category and the average survival time that they have in the following years. In particular, the analysis shows that 40% of new terms are basically small variations of terms already known. Only two of them stand out for their great acceptance in the subsequent years, 'POSTURAL BALANCE' and 'DOUBLE BUNDLE'. Although there are no similar studies with which we can compare our data, it seems that the area has a good attitude towards the new words. However, they have scarce relevance since more than half of the AKs fail to pass the first year. According to our survival analysis, the average lifetime for a new word is 3 years. Only 9% of the words were used throughout the survival period. The works of Santos and Irizo [68,69] employ a model of analysis closer to ours, using the citations received by the articles. Obviously, the behaviour of citations and keywords does not have to be similar; however, we have found some similarities. Although the results section has simplified the analyses carried out to improve reading fluency, like Santos and Irizo we have tested our empirical model with different theoretical models. As with their findings, the distribution that best fits here is the Weibull distribution (k = 0.69, SE = 0.01, where k is the shape parameter), which indicates that the failure rate decreases over time. In other words, despite the sharp decline of the first year, data indicate that after a while, words begin to gain strength in the SSC.

The main limitations of our work are related to the methodology used. In our research, we choose AKs as an indicator of the contents that appear in the articles, but authors may not properly select them. Moreover, AKs from the SSC present some inaccuracies in the way that they are written, since some of them show an excessive extension. However, the impact of these singularities on our results is diluted since we have analysed the entire universe of AKs that have appeared within the WOS category. Works that choose smaller samples will have to take this limitation into account.

A second limitation refers to the period of AK analysis of 40 years and not since the beginning of the SSC. The studies whose objectives include the historical evolution of concepts should necessarily opt for the use of other fields of search in the database. The third is about the survival analysis that is restricted to the appearance of AKs in the SSC, although the words could appear in a different WOS category or other parts of the paper (title, abstract or main text).

Finally, the way in which the results are shown in this article (ordered by their frequency of appearance) may highlight the most common topics but not the most important ones. To save space, the tables and figures of our article only contain those terms that reached a high number of repetitions and excluded those terms that are more residual or less frequent from a quantitative point of view. This method limits our results, probably because the front of the SSC knowledge is made of AKs with low or medium frequency. For this reason, we choose to increase the information of this paper and make it available to the scientific community in the supplementary materials for future interpretations and analyses. Despite the effort made by the research team to maintain a neutral tone in the discourse of this article, our own background as researchers may have influenced the way of ordering and discussing the results. This is especially relevant in the case of a global analysis such as the one presented in this paper. Future studies should discuss our results from the point of view of researchers from other disciplines or people who, because of their professional work (e.g., journal editors), have a global but different view of the Sport Sciences field of study.

## Conclusions

One of the main findings of our research is the identification of 6 large thematic clusters in the SSC. There are also two major terms that coexist ('REHABILITATION' and 'EXERCISE') and show high frequencies of appearance, as well as a key behaviour in the calculated co-occurrence networks. Another significant finding is that new AKs are mostly accepted in the SSC since a high percentage of new terms during 2001–2006 were observed, although with a low survival period. These results support a multidisciplinary perspective within the Sport Sciences field of study and a colonization of the field by rehabilitation according to our AK analysis. This global view of the SSC has been possible through the methodology used, which includes data mining methods for the analysis of a large amount of data. Of special interest is the survival analysis developed because it represents a new methodology in the AKs analysis. This type of analysis opens new possibilities in different areas of research to study trends and introduction of new words, not only in the academic world but also in the information and communication professional contexts.

## Supporting information

S1 File ISSN. (PDF)

S1 Table KW frequency. (XLSX)

S2 Table Centrality parameters. (XLSX)
