# Supplementary material for: An author keyword analysis for mapping Sport Sciences
Source: PLoS One. 2018 Aug 1;13(8):e0201435. doi: 10.1371/journal.pone.0201435 (PMC6070281; doi:10.1371/journal.pone.0201435)
Supplement: S1 File — (PDF) [file pone.0201435.s001.pdf]

IS=(1077-5552 OR 0112-1642 OR 0306-3674 OR 0363-5465 OR 0091-6331 OR 0195-9131 OR 1440-2440 OR 8750-7587 OR 0905-7188 OR 0003-9993 OR 0942-2056 OR 0190-6011 OR 1469-0292 OR 1058-2746 OR 1746-1391 OR 1555-0265 OR 0264-0414 OR 2095-2546 OR 0895-2779 OR 0966-6362 OR 1062-6050 OR 0890-5339 OR 1050-642X OR 1439-6319 OR 1526-484X OR 0172-4622 OR 1550-2783 OR 1064-8011 OR 1715-5312 OR 0968-0160 OR 0268-0033 OR 0167-9457 OR 1063-8652 OR 1466-853X OR 0270-1367 OR 1543-8627 OR 1303-2968 OR 1934-1482 OR 0894-9115 OR 1527-0297 OR 0899-8493 OR 1650-1977 OR 1050-6411 OR 1643-8698 OR 0278-5919 OR 0736-5829 OR 0860-021X OR 1056-6716 OR 1041-3200 OR 0033-6297 OR 1062-8592 OR 1357-3322 OR 0022-2895 OR 1537-890X OR 0091-3847 OR 0888-4773 OR 0888-4781 OR 0022-4707 OR 1065-8483 OR 1754-3371 OR 1331-1441 OR 0095-6562 OR 1476-3141 OR 1640-5544 OR 1087-1640 OR 1474-8185 OR 1080-6032 OR 0273-5024 OR 0741-1235 OR 0047-0767 OR 1728-869X OR 1091-5397 OR 1524-1602 OR 0765-1597 OR 1577-0354 OR 0932-0555 OR 1517-8692 OR 0940-6689 OR 0959-3020 OR 0025-7826 OR 1060-1872)
